# Developing a synthetic psychosocial stress measure and harmonizing CVD-risk data: a way forward to GxE meta- and mega-analyses

**DOI:** 10.1186/s13104-018-3595-z

**Published:** 2018-07-24

**Authors:** Abanish Singh, Michael A. Babyak, Beverly H. Brummett, William E. Kraus, Ilene C. Siegler, Elizabeth R. Hauser, Redford B. Williams

**Affiliations:** 1 Behavioral Medicine Research Center, Duke University School of Medicine, Durham, NC USA; 2 Department of Psychiatry and Behavioral Sciences, Duke University School of Medicine, Durham, NC USA; 3 Duke Molecular Physiology Institute, Duke University School of Medicine, Durham, NC USA; 4 Department of Medicine, Duke University School of Medicine, Durham, NC USA; 5 Department of Biostatistics and Bioinformatics, Duke University School of Medicine, Durham, NC USA

**Keywords:** Data harmonization, GxE interaction, CVD-risk, Mega-analysis, Synthetic psychosocial stress, Depressive symptoms, Correlation

## Abstract

**Objectives:**

Among many challenges in cardiovascular disease (CVD) risk prediction are interactions of genes with stress, race, and/or sex and developing robust estimates of these interactions. Improved power with larger sample size contributed by the accumulation of epidemiological data could be helpful, but integration of these datasets is difficult due the absence of standardized phenotypic measures. In this paper, we describe the details of our undertaking to harmonize a dozen datasets and provide a detailed account of a number of decisions made in the process.

**Results:**

We harmonized candidate genetic variants and CVD-risk variables related to demography, adiposity, hypertension, lipodystrophy, hypertriglyceridemia, hyperglycemia, depressive symptom, and chronic psychosocial stress from a dozen studies. Using our synthetic stress algorithm, we constructed a synthetic chronic psychosocial stress measure in nine out of twelve studies where a formal self-rated stress measure was not available. The mega-analytic partial correlation between the stress measure and depressive symptoms while controlling for the effect of study variable in the combined dataset was significant (*Rho *= 0.27, *p *< 0.0001). This evidence of the validity and the detailed account of our data harmonization approaches demonstrated that it is possible to overcome the inconsistencies in the collection and measurement of human health risk variables.

**Electronic supplementary material:**

The online version of this article (10.1186/s13104-018-3595-z) contains supplementary material, which is available to authorized users.

## Introduction

Psychosocial stress, defined as aversive or demanding environmental conditions that exceed the resources of an organism, has often been implicated in the genesis of cardiovascular disease (CVD) and CVD-risk factors [1, 2]. Stress also may play a significant role in modifying the impact of genetic factors on CVD-risk [3, 4]. A better understanding of how genes interact with stress to contribute to CVD pathways might be gained by developing robust estimates of gene-by-stress interactions across the disease pathways in the context of genetic variations and demographic differences.

Detecting and generalizing statistical interaction typically requires considerably larger sample sizes than needed for statistical main effects [5]. Moreover, some interactions are observable only in limited situations and may not be broadly generalizable [6]. One approach to overcoming these challenges is to exploit the large accumulation of epidemiological data, thereby increasing sample size and statistical power. These data can be used to conduct a conventional *meta*-*analysis*, a potential new standard of original research [7], in which an aggregate estimate is generated using summary statistics from individual new studies or already reported in the literature [8]. Alternatively, the individual-level data can be combined into a single harmonized dataset upon which new analyses are carried out. This latter approach is often referred to as *mega*-*analysis* [8].

Studies with inconsistent measurements, protocols, and methods may lead to inconsistent conclusions. Moreover, the integration of measures across studies with heterogeneous measurement protocols, units and coding can be a significant challenge. This challenge has resulted in several data harmonization efforts [9–11], but these have focused mostly on the design and standardization of measures for use in future studies. A more significant challenge is the circumstance in which there may not be an explicit measure of the phenotype of interest in existing studies. In pursuing our work on psychosocial stress, we were immediately confronted by the absence of an explicit measure of psychosocial stress in many studies. Thus, an important undertaking in our previous work was to develop an algorithm for constructing a valid measure of psychosocial stress from extant datasets with no explicit stress measure [4]. We refer to this as a “synthetic” measure of psychosocial stress to distinguish it from formal self-rated measures developed specifically to assess stress. Our synthetic stress algorithm is based on the items of the formal, self-rated measure of chronic psychosocial stressors known as “chronic burden” in the Multi-Ethnic Study of Atherosclerosis (MESA) [12]. The MESA chronic burden measure, as well as similar indicators of stress, are associated with a range of CVD-risk factors [3, 13–16].

In the present paper, we provide a detailed illustration of a number of decisions made in the process of creating the synthetic stress measure, the harmonization of inconsistency among CVD-risk variables, and subsequent combining the harmonized data from a dozen different studies into a single dataset. We also provide a mega-analytic estimate of association between stress and depressive symptoms. The harmonized data matrix also included single nucleotide polymorphisms (SNPs) *EBF1* rs4704963, *5HTR2C* rs6318, and *BDNF* rs6265, which we found associated with CVD-risk factors in the presence of stress in our earlier work [3, 17, 18]. These efforts will allow us to develop robust estimates of gene-by-stress interactions.

## Main text

### Methods and material

#### Data sources

We used a dozen (six dbGaP and six Duke) datasets in this data harmonization study. The dbGaP public-access datasets were from the Women’s Health Initiative (WHI) Study [19]; Coronary Artery Risk Development in Young Adults Study (CARDIA) [20]; Atherosclerosis Risk in Communities Study (ARIC) [21]; Framingham Offspring Cohort [22]; Multi-Ethnic Study of Atherosclerosis (MESA) [23]; and Jackson Heart Study (JHS) [24]. The Duke datasets were from the Community Health and Stress Evaluation (CHASE) Study [25]; Duke Family Heart Study (DFHS) [26]; Duke Caregiver Study (DCS) [27]; and three cohorts for Studies of a Targeted Risk Reduction Intervention through Defined Exercise (STRRIDE), i.e., STRRIDE I [28], STRRIDE–Aerobic Training/Resistance Training (AT/RT) [29], and STRRIDE Pre-Diabetes (PD) [30] studies. A brief description of the contributing studies is provided in the Additional file 1.

#### Building a synthetic stress measure

Using the algorithm described in [4], we constructed our synthetic stress measure in four out of six dbGaP datasets and five out of six Duke datasets where a self-rated formal stress measure was not available. The MESA and JHS datasets included a self-rated stress measure. In the absence of items that specifically query about stress, the algorithm uses proxy indicators of the domains used in the MESA chronic burden measure [12]: financial strains, relationship or marital problems, difficulties with job or ability to work, serious health problems of spouse or someone close, and one’s own serious health problems. The steps of our algorithm [4] included searching the most suitable proxy item for as many of the five components as possible, scoring them as 0 or 1 using the proxy item, and creating the synthetic Singh et al. [4] chronic stress ordinal variable by summing all available binary components. Our analysis in previous work suggested that a synthetic variable developed using incomplete set of two (worst case), three, or four proxy items could still be useful, when all five proxy items were not available.

*Pseudocodes* We provide a description and pseudocodes for the construction of synthetic stress measure in all studies in the Additional file 1.

#### Validation of synthetic stress measure

Assuming that the samples under study were at least broadly similar culturally, we evaluated the distributions of the synthetic stress measure in each dataset and compared them with available self-rated measures, and expected the shape of distributions to be reasonably similar across studies. We provided additional support for the validity of the synthetic stress measure by evaluating its well-known association (Spearman correlation) with a measure of depressive symptoms.

*Mega*-*analysis* We also estimated a partial correlation of stress and depressive symptoms in harmonized, combined data whilst controlling for the effect of study dummy variables.

#### Additional steps in data harmonization

*Harmonizing variability in units and coding* For phenotype measures that were presented in different units across the studies, we used accepted conversion factors (Additional file 1: Table S1) to create a corresponding single unified variable. The inconsistent codings for sex and race were also reconciled. Ordinal measures of a phenotype that differed in terms of the number of possible responses (e.g., chronic stress, depressive symptoms) were converted to *z*-scores (SD = 1, mean = 0) within each study.

*Accounting for data sources* In order to facilitate the mega-analysis using combined multiple datasets, we created vectors of dummy indicators for each study. These variables enable adjustment for study origin and as possible effect modifiers in fixed effects models. More details on the choice of dummy variable over random effects coding are provided in Additional file 1.

*Dealing with outliers* Extreme outlying values can unduly influence statistical estimates of association or central tendency and are typically excluded or trimmed to less extreme values. One potential challenge in this regard is that it is not always possible to determine whether outliers are the actual measured values or the result of errors. More details on outlier detection and removal are provided in Additional file 1.

*Summary statistics* Finally, we evaluated summary statistics and distributions of harmonized data variables in order to evaluate consistency in the harmonized measurements and differences across the study cohorts.

#### Genetic data: identifying proxy SNPs

We harmonized the candidate SNPs of interest (rs4704963, rs6318, and rs6265, as reviewed above) across all datasets. We identified proxy SNPs for a missing SNP using two criteria (1) a high score of the proxy SNP with the SNP of interest (Linkage Disequilibrium R^2^ ≥ 0.95) and (2) availability of same proxy SNP in each dataset. The SNP data for each study was subjected to a standard quality control before selecting the candidate SNPs (Additional file 1).

### Results

**Fig. 1:**
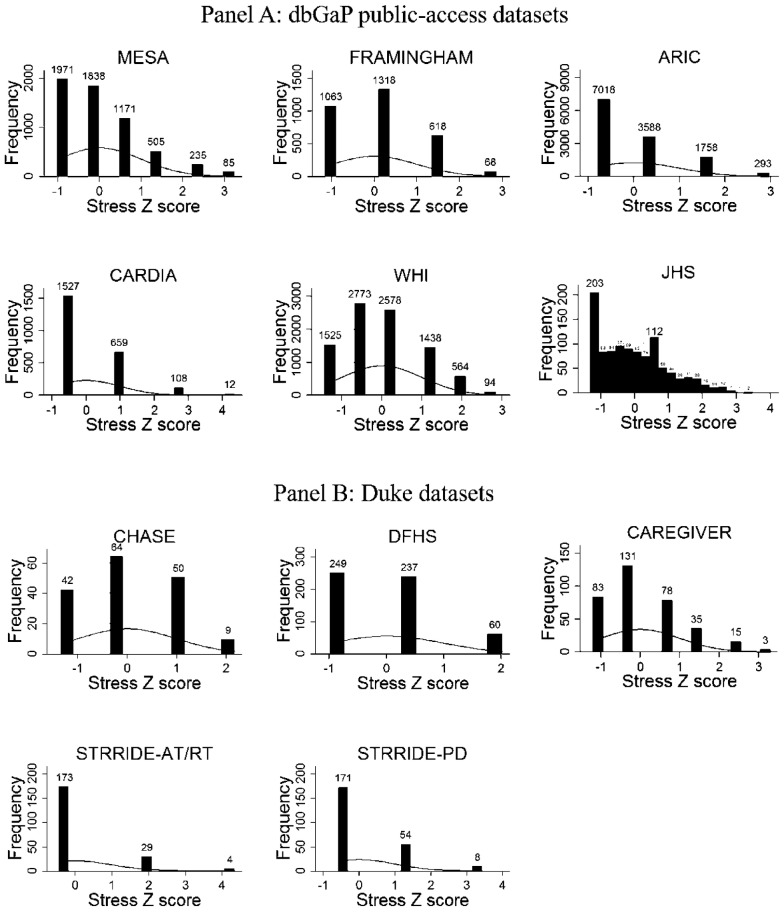
**a** Distributions of chronic stress *z*-scores in dbGaP public-access datasets, i.e., MESA, Framingham Offspring, ARIC, CARDIA, WHI and JHS, and **b** Duke datasets, i.e., CHASE, DFHS, Duke Caregiver, STRRIDE-AT/RT, and STRRIDE-PD. With the exception of MESA and JHS, the stress measure is a synthetic variable for all datasets

The distribution of the chronic psychosocial stress *z*-scores are presented in Fig. 1. The synthetic stress variable appears for all datasets, with the exception of MESA, which used the aforementioned chronic burden measure, and JHS, which, in addition to the five domains, also assessed stress due to legal problems, racism/discrimination, and neighborhood characteristics. The similarity in shapes of z-scores distributions (i.e., flat, skewed toward the right; kurtosis = 2.19–7.92, skewness = 0.20–2.34) adds additional support to our contention that the synthetic stress was assessing a similar underlying construct in different studies.

The Spearman correlations of the synthetic measures (Table 1) for all datasets except CARDIA (*Rho *= 0.07) were reasonably strong (*Rho *= 0.20–0.57), significantly different from zero (*p *< 0.001), and similar in magnitude to those observed for the self-rated measures (i.e., MESA and JHS). Some of the possible reasons for the weak correlation in CARDIA may be due to the facts that the available CES-D depression measure was assessed in a later exam that followed baseline and that it was the youngest cohort (mean age 24.97 years). Controlling for the effect of study variable, mega-analytic partial correlation between stress and depression in combined dataset was significant (*Rho *= 0.27, *p *< 0.0001). As expected, the significant correlations between the measures of synthetic stress and depressive symptoms in all datasets except one further supports the validity of our method for the construction of synthetic stress measures in datasets that lacked a self-rated measure.

**Table 1:** Spearman’s correlation of synthetic and self-rated stress measures with CES-D depression measure

Dataset	Stress measure	Depression measure	*Rho*	P-value
MESA	Self-rated	Center for Epidemiological Studies-Depression Scale (CES-D)	0.35	< 0.0001
Framingham Offspring Cohort	Synthetic	CES-D	0.23	< 0.0001
CARDIA	Synthetic	CES-D	0.07	< 0.001
ARIC	Synthetic	Maastricht Vital Exhaustion Score	0.28	< 0.0001
WHI	Synthetic	Shortened CES-D	0.2	< 0.0001
JHS	Self-rated	CES-D	0.32	< 0.0001
CHASE	Synthetic	Beck Depression Inventory (BDI)	0.31	< 0.001
DFHS	Synthetic	CES-D	0.25	< 0.0001
Caregiver	Synthetic	CES-D	0.42	< 0.0001
STRRIDE-AT/RT	Synthetic	Self-rated	0.4	< 0.0001
STRRIDE-PD	Synthetic	Self-rated	0.54	< 0.0001
Combined estimate^a^			0.27	< 0.0001

^a^ Combined estimate derived from mega-analytic partial correlation between standardized (z-scores) stress and depression measures whilst controlling for the effect of study variable in combined dataset

**Fig. 2:**
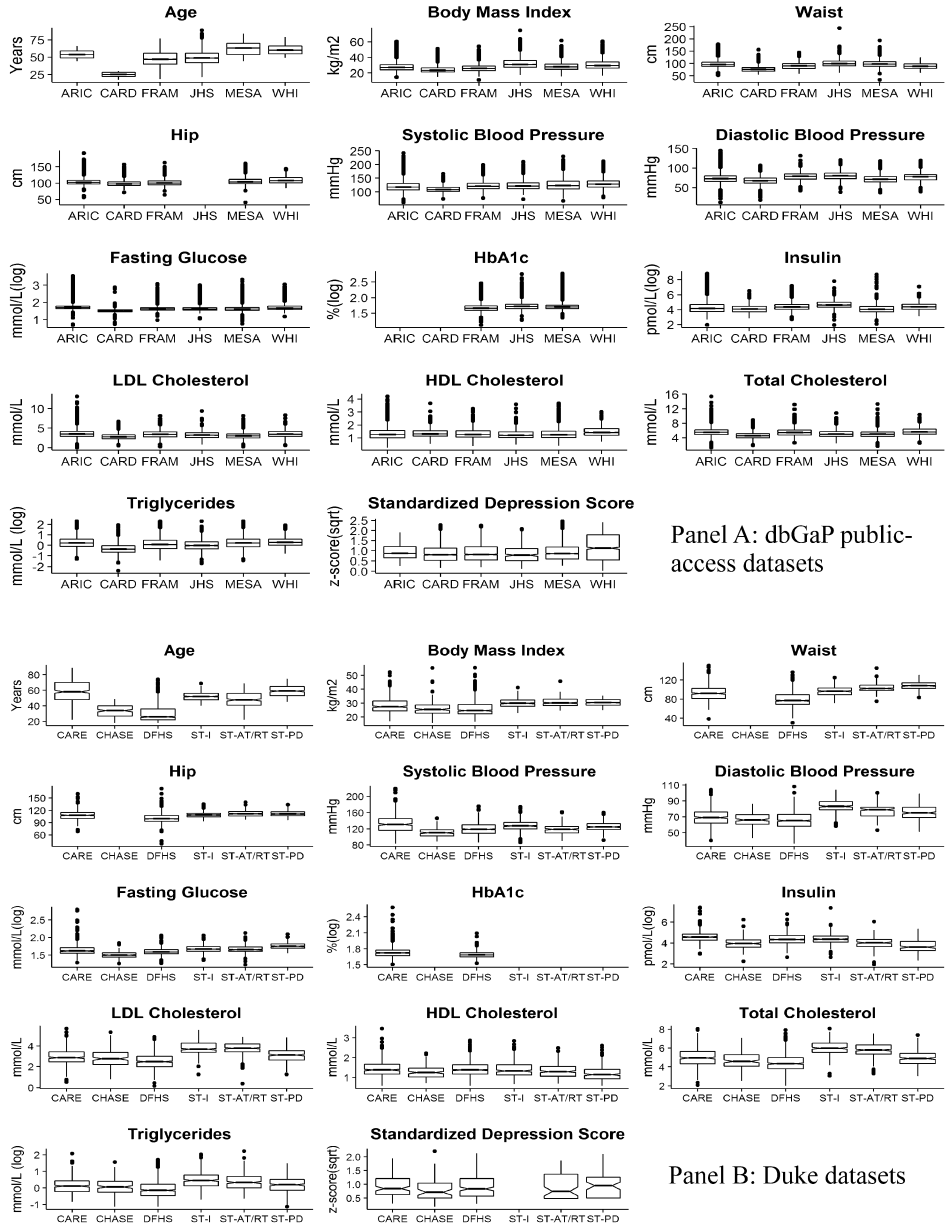
**a** Distributions of harmonized phenotypes in dbGaP public-access datasets. Each notched box plot shows the distribution (i.e., five point summary statistics, outliers, and notches based on the median ± 1.58 * IQR/sqrt(n)) of one variable in the six dbGaP studies, i.e., ARIC, CARDIA, FRAMINGHAM, JACKSON HEART, MESA, and WHI; and **b** six Duke studies, i.e., CAREGIVER, CHASE, DFHS, STRRIDE-1, STRRIDE-AT/RT, and STRRIDE-PD. The scales for fasting glucose, insulin, HbA1C, and triglyceride were log transformed, and the standardized depression measure was square root transformed

Although the units for blood pressure, BMI, and age were consistent across the datasets, studies differed in the units of other measures such as fasting glucose, insulin, and lipids (Additional file 1: Table S2 Panel A and B). The codings for sex and race were also inconsistent across the datasets. Finally, the CES-D depression measure was also scored differently in Framingham Heart Study (range 0–0.85) as compared to other datasets and a shortened version of the CES-D was used in WHI, which were converted to z-scores. While the uniformity in the summary statistics of harmonized variables (Additional file 1: Table S3) support the tenability of the harmonization process, they also demonstrate the underlying differences in each cohort in terms of age and CVD-risk factors. The distribution plots of each CVD-risk variable for each datasets (Fig. 2a, b) provide a comparison of harmonized measurements across all the datasets and document the consistency of our harmonization approaches.

The harmonization of three SNPs, which moderated the influence of stress on CVD-risk endophenotypes in our prior research, resulted in proxy SNPs in the place of missing SNPs in dataset(s) with perfect LD score (R^2^ = 1.0). The minor allele frequency (MAF) differences among the Whites and Blacks suggested that race-stratified analysis of genetic association might be preferred for these SNPs (Additional file 1: Table S4).

### Discussion

In our previous work [4], we provided an algorithm to construct synthetic stress measure and a systematic comparison of a synthetic and self-rated measure with evidences for unidimensionality using the MESA dataset. In the present work, we describe the details of our undertaking to harmonize twelve datasets and we provide the set of proxy indicators and pseudocodes for constructing synthetic stress in nine out of twelve studies in hope that it will help the scientific community in further work. Two studies (MESA, JHS) had a formal self-rated stress measure and one study (STRRIDE-I) did not have any proxy indicator for synthetic measure. The construction of a synthetic stress measure in datasets that did not have a self-rated formal stress measure is a key innovation in the current study. The broad domains of psychosocial stress that we have used in our synthetic stress construction algorithm [4] have been frequently used as part of formal stress measures [12, 31]. Thus, our synthetic stress measure is consistent with that of others using similar stress domains, which are apparently sufficient to capture life stress, even when not all present [4]. Past research has found one or more of these domains to be associated with a number of CVD-risk factors, such as, glucose metabolism [32], blood pressure [33], mortality [34], cortisol [35], and depressive symptoms [36]. Another important aspect of our work was to obtain insights from large sample resulting from combining the datasets. Summarizing results over multiple studies, either through conventional meta-analysis, or as in our case using mega-analysis (Table 1), is thought to produce a more robust estimate of the associations under study, and potentially more generalizable insights [37]. We provide additional discussion in the Additional file 1.

### Conclusions

We illustrated our method used to construct a synthetic stress measure with evidences of its validity. Our work provides ways by which to harmonize and operationalize the existing data and overcome the inconsistencies in the collection and measurement of human health risk variables that we hope will complement and support other ongoing efforts to standardize measurements in new studies. This works also provides the opportunity of future work to perform more robust and informative mega-analytic tests of our prior findings on gene-by-stress interactions modifying expression of endophenotypes in CVD pathways.

## Limitations

We have so far chosen not to impute for missing variables and indicators in our approach. This choice is supported by our prior work [4] showing that the stress scores which include less than the full set of indicators still behave similarly in terms of associations with other phenotypes, such as depressive symptoms. A formal measure designed explicitly to assess stress would generally be the most desirable choice to use in investigations; however, when the formal measure is not available, the five-component synthetic stress measure appears to serve as an acceptable utilitarian solution.

## Additional file

**Additional file 1.** The additional file provides more details on the data sources of contributing studies; proxy indicators and pseudocodes for synthetic stress measure; SNP quality control; coding for studies from multiple sources; outlier detection and removal; and additional discussion on the need for data harmonization, harmonization steps and alternative approaches, and insights from large sample resulting from combining the datasets.

## Abbreviations

CVD: cardiovascular disease
GxE: gene-by-environment interaction
SNP: single nucleotide polymorphism
*EBF1*: early B-cell factor 1
*BDNF*: brain-derived neurotrophic factor
*5HTR2C*: 5-hydroxytryptamine receptor 2C
dbGaP: database of genotypes and phenotypes
WHI: Women’s Health Initiative Study
CARDIA: Coronary Artery Risk Development in Young Adults Study
MESA: Multi-Ethnic Study of Atherosclerosis
JHS: Jackson Heart Study
CHASE: Community Health and Stress Evaluation Study
DFHS: Duke Family Heart Study
DCS: Duke Caregiver Study
STRRIDE I: Study of a Targeted Risk Reduction Intervention through Defined Exercise (cohort I)
STRRIDE–AT/RT: Study of a Targeted Risk Reduction Intervention through Defined Exercise–Aerobic Training/Resistance Training
STRRIDE–PD: Study of a Targeted Risk Reduction Intervention through Defined Exercise–Pre-Diabetes
BDI: Beck Depression Inventory
CES-D: Center for Epidemiological Studies-Depression Scale
