# Supplementary material for: Developing a synthetic psychosocial stress measure and harmonizing CVD-risk data: a way forward to GxE meta- and mega-analyses
Source: BMC Res Notes. 2018 Jul 24;11:504. doi: 10.1186/s13104-018-3595-z (PMC6057001; doi:10.1186/s13104-018-3595-z)
Supplement: Supplementary file 1 — Additional file 1. The additional file provides more details on the data sources of contributing studies; proxy indicators and pseudocodes for synthetic stress measure; SNP quality control; coding for studies from multiple sources; outlier detection and removal; and additional discussion on the need for data harmonization, harmonization steps and alternative approaches, and insights from large sample resulting from combining the datasets. [file 13104_2018_3595_MOESM1_ESM.docx]

Additional file

Developing a synthetic psychosocial stress measure and harmonizing CVD-risk data: A way forward to GxE meta- and mega-analyses

Abanish Singh^*, 1, 2, 3^, Michael A. Babyak^1,2^, Beverly H. Brummett^1,2^, William E. Kraus^3,4^, Ilene C. Siegler^1,2^, Elizabeth R. Hauser^3,5^ and Redford B. Williams^1,2^

^1^Behavioral Medicine Research Center, Duke University School of Medicine, Durham, NC, USA; ^2^Department of Psychiatry and Behavioral Sciences, Duke University School of Medicine, Durham, NC, USA; ^3^Duke Molecular Physiology Institute, Duke University School of Medicine, Durham, NC, USA; ^4^Department of Medicine, Duke University School of Medicine, Durham, NC, USA; ^5^Department of Biostatistics and Bioinformatics, Duke University School of Medicine, Durham, NC, USA

*Correspondence: Abanish Singh, Ph.D.

Duke University School of Medicine

Duke Box 104775, Durham, NC  27701

Tel: +1 919 681 2133 Fax: +1 919 684 0934

Email: abanish.singh@duke.edu

**Data Sources**

The public access datasets we used were obtained from the data depository dbGaP/database of Genotypes and Phenotypes/ National Center for Biotechnology Information, National Library of Medicine (NCBI/NLM)/https://www.ncbi.nlm.nih.gov/gap through an authorized access. The in-house datasets were obtained from our Duke in-house studies. The studies are as follows.

***The Women’s Health Initiative (WHI):*** WHI is a long-term national health study dedicated to developing prevention strategies for heart disease, breast and colorectal cancer, and osteoporotic fractures in postmenopausal women [1]. The groundbreaking results from this study have made significant contributions towards the care and prevention of some of the major health conditions affecting postmenopausal women.

***The Coronary Artery Risk Development in Young Adults Study (CARDIA):*** CARDIA was designed to study the etiology and natural history of cardiovascular disease beginning in young adulthood [2]. The CARDIA study participants’ selection constituted approximately the same number of people in subgroups of race, gender, education.

***Atherosclerosis Risk in Communities Study (ARIC):*** ARIC is a prospective epidemiologic study focused to investigate the etiology and natural history of atherosclerosis and demographic variation in cardiovascular risk factors, medical care, and disease [3]. The study examined atherosclerosis by direct observation and by use of modern biochemistry. The components of the study included identification, investigation, and diagnosis of clinical events through home interviews, clinic examinations, and annual telephone follow-ups.

***Framingham Offspring Cohort:*** We used the Generation 2 (or Offspring) dataset from the Framingham Heart Study Cohort for this work [4] because of availability of psychosocial measurements and genetic data. The second-generation cohort included adult children (and their spouses) of the original participants. The cohort is primarily White.

***Multi-Ethnic Study of Atherosclerosis (MESA):*** MESA was designed to study the CVD risk factors that predict progression of the clinically observable or subclinical cardiovascular disease [5]. The dataset included a well-characterized self-rated chronic psychosocial stress summary measure “chronic burden", quantified on an ordinal scale of 0 to 5 (0, 1, 2, 3, 4, and 5) based on questionnaires in five domains including questions about ongoing serious health problems, serious health problems with someone close, work-related problems, financial strains, and difficulties in relationships [6-8].

***Jackson Heart Study (JHS)***: The JHS is a large, community-based, observational study that was designed to explore reasons for the prevalence of cardiovascular disease among African Americans [9]. The study participants were recruited from urban and rural areas of the Jackson MS, metropolitan statistical area (MSA). The study aimed to identify genetic factors that affect cardiometabolic risk factors in African Americans. The study variables included a self-rated psychosocial stress summary measure that was based on eight items, i.e., stress due to job, relationships, neighborhood, care-giving, legal problems, medical problems, racism/discrimination, and (not) meeting basic needs [10].

***Community Health and Stress Evaluation (CHASE) Study:*** The Duke CHASE Study was designed to determine the role of psychosocial and biobehavioral factors in the etiology of coronary heart disease. The study was based an invasive medical procedure (a spinal tap) and the medical information survey in varying socioeconomic status (SES) of Black and White groups [11].

***Studies of a Targeted Risk Reduction Intervention through Defined Exercise (STRRIDE):*** The Duke STRRIDE cohort includes three studies: STRRIDE I, STRRIDE – Aerobic Training / Resistance Training (AT/RT), and STRRIDE pre-diabetes (PD). The purpose of STRRIDE I study was to investigate the peripheral biological mechanisms through which chronic physical activity altered carbohydrate metabolism and lipid metabolism [12]. STRRIDE AT/RT study was designed to compare the effects of aerobic training (AT) and resistance training (RT) and the full combination (AT/RT) on central ectopic fat and liver enzymes and fasting insulin resistance by homeostatic model assessment (HOMA) [13]. The purpose of the STRRIDE-PD study was to compare the effects of different amounts and intensities of exercise training programs without diet to an exercise and diet program modeled after the first six months of the Diabetes Prevention Program (DPP) [14].

***Duke Caregiver Study (DCS):*** This study was conducted at the Duke University Medical Center that included data from family caregivers of a relative with Alzheimer’s disease or other dementia and non-caregiving control [15].

***Duke Family Heart Study (DFHS):*** This study was conducted at Duke University Medical Center under the approval of Duke IRB to study the effect of genetic variation on the relationship between psychosocial and cardiovascular risk factors [16].

**Proxy Indicators and Pseudocodes:**

In the absence of a formal self-rated stress measure in a dataset, our synthetic stress algorithm [6] uses proxy indicators of the five components used in the MESA chronic burden measure [8]. These components are following: financial strains (i.e., financial stress component, FSC), relationship or marital problems (i.e., marital stress component, MSC), difficulties with job or ability to work (i.e., work stress component, WSC), serious health problems of spouse or someone close (i.e., health other’s stress component, HOSC), and one’s own serious health problems (i.e., health stress component, HSC). We attempted to identify the proxy indicators for as many of the five components (i.e., FSC, MSC, WSC, HOSC, and HSC) as possible in each datasets. The description of proxy indicators and pseudocodes used to create synthetic Singh *et al.* chronic stress (SCS) ordinal variable in each dataset is provided as follows.

In order to create a synthetic measure of psychosocial stress in WHI, we used marital status, income, work difficulties (wrkdiff), general health (gebhel), and help sick (hlpsick) to derive components related to stress due to relationship or marital problems, financial strains, work related stress, stress due to own health problems, and stress due to health of someone close, respectively. The pseudocode for creating the synthetic stress variable in the WHI is below:

*MSC = 1 if marital== DIVORCED OR SEPARATED;*

*MSC = 0 if MSC == NULL AND marital != NULL;*

*FSC = 1 if income < MEDIAN INCOME;*

*FSC = 0 if income >= MEDIAN INCOME AND income != DON’T KNOW;*

*WSC = 1 if wrkdiff ==YES;*

*WSC = 0 if wrkdiff ==NO;*

*HSC = 1 if genhel == Fair OR Poor;*

*HSC = 0 if genhel == Excellent OR Very Good OR Good;*

*HOSC = 1 if hlpsick==YES;*

*HOSC = 0 if hlpsick==NO;*

*SCS = MSC+FSC+WSC+HSC+HOSC if no more than one component missing;*

To construct a synthetic measure of psychosocial stress in CARDIA, we identified the following proxy items “OTHER MAJOR DISEASES OR HEALTH PROBLEMS" (hlthp), “EXPECT TO REACH FINANCIAL SUCCESS" (finan), “MARITAL STATUS" (marital), “FIRED FROM A JOB" (fire), “GOT DEMOTED AT WORK" (demot), and “LAID OFF FROM A JOB" (laid). These items were then used to derive HSC, FSC, MSC, and WSC components of synthetic stress measure. The pseudocode for creating the synthetic stress variable in the CARDIA is below:

*HSC = 0 if helthp== NO;*

*HSC = 1 if helthp== YES;*

*FSC = 0 if finan == YES";*

*FSC = 1 if finan == NO;*

*MSC = 1 if marital == DIVORCED OR SEPARATED;*

*MSC = 0 if MSC = NULL AND (marital != REFUSED OR DON’T KNOW);*

*WSC = 0 if demot == NO OR fire == NO OR laid ==NO;*

*WSC = 1 if demot == YES OR fire == YES OR laid == YES;*

*SCS = MSC+FSC+WSC+HSC if no more than one component missing;*

In ARIC to construct a synthetic measure of psychosocial stress, we used income, health compare to other people of same age, and marital status to derive the FSC, HSC, and MSC. The pseudocode for creating the synthetic stress variable in the ARIC is below:

*MSC = 1 if marital== DIVORCED OR SEPARATED;*

*MSC = 0 if MSC == NULL AND marital != NULL;*

*FSC = 1 if income < MEDIAN INCOME;*

*FSC = 0 if income >= MEDIAN INCOME;*

*HSC = 1 if health == Fair OR Poor;*

*HSC = 0 if health == Excellent OR Good;*

*SCS = MSC+FSC+HSC if no more than one component missing;*

In CHASE we used proxy items marital status, household income, overall health, and the work related difficulties (wrkdiff) that was scored based on questions assessing job security, lack of career prospects, issues with support at work (supervisor and others), and job dissatisfaction. The pseudocode for creating the synthetic stress variable in the CHASE is below:

*MSC = 1 if marital== DIVORCED OR SEPARATED;*

*MSC = 0 if MSC == NULL AND marital!=NULL;*

*FSC = 1 if income < MEDIAN INCOME;*

*FSC = 0 if income >= MEDIAN INCOME;*

*WSC = 0 if wrkdiff <= MIDPOINT OF wrkdiff SCORE;*

*WSC = 1 if wrkdiff > MIDPOINT OF wrkdiff SCORE;*

*HSC = 1 if health == Fair OR Poor;*

*HSC = 0 if health == Excellent OR Very Good OR Good;*

*SCS = MSC+FSC+WSC+HSC if no more than one component missing;*

In STRRIDE, we identified proxy items: “In general, would you say your health is ...” (GENHLTH) for the self-health problems component (i.e., HSC) and “Accomplished less than you would like …” (i.e., job demand, ACOMPLESS) for the difficulties with job or ability to work component (i.e., WSC). The pseudocode for creating the synthetic stress variable in the STRRIDE is below:

*HSC = 0 if GENHLTH == Excellent OR Very Good OR Good;*

*HSC = 1 if GENHLTH == Fair OR Poor;*

*WSC = 0 if ACOMPLESS == A little of the time OR None of the time;*

*WSC = 1 if ACOMPLESS == All of the time OR Most of the time OR Some of the time;*

*SCS = HSC + WSC if HSC != NULL AND WSC !=NULL*

Although we reported the construction of synthetic stress variable for following datasets (i.e., Framingham Offspring, Caregiver, and DFHS) in our previous work [6], we included these datasets again in this work to provide harmonization of CVD-risk variables and a comprehensive comparison of synthetic stress measure. We provide the pseudocodes for the construction of synthetic stress measure in these datasets below:

In Duke Caregiver Study, we used proxy items household income, spouse related hassles, hassles related to health or well-being of a family member, and self-health related hassles for the indicators of financial strain, marital problems, health problems of spouse or someone close, and one’s own serious health problems components, respectively. We used a score of hassles related to job security, meeting deadlines or goals (i.e., job demand), and hassles related to supervisor and fellow workers (i.e. support at work) for the proxy indicators of work difficulties component [6]. The pseudocode for creating the synthetic stress variable in this study is below:

*MSC = 1 if marital== DIVORCED OR SEPARATED;*

*MSC = 0 if MSC == NULL AND marital!=NULL;*

*FSC = 1 if income < MEDIAN INCOME;*

*FSC = 0 if income >= MEDIAN INCOME;*

*WSC = 0 if wrkdiff <= MIDPOINT OF wrkdiff SCORE;*

*WSC = 1 if wrkdiff > MIDPOINT OF wrkdiff SCORE;*

*HSC = 1 if health == Fair OR Poor;*

*HSC = 0 if health == Excellent OR Very Good OR Good;*

*SCS = MSC+FSC+WSC+HSC if no more than one component missing;*

In DFHS, we identified total income for the indicator of the financial strain component; and the work difficulties component was scored based on 10 questions on job insecurity, lack of career prospects, issues with support at work (supervisor and others), and job dissatisfaction (i.e., a total two components out of five) [6]. The pseudocode for creating the synthetic stress variable in the DFHS is below:

*FSC = 1 if income < MEDIAN INCOME;*

*FSC = 0 if income >= MEDIAN INCOME;*

*WSC = 0 if wrkdiff <= MIDPOINT OF wrkdiff SCORE;*

*WSC = 1 if wrkdiff > MIDPOINT OF wrkdiff SCORE;*

*HSC = 1 if health == Fair OR Poor;*

*HSC = 0 if health == Excellent OR Very Good OR Good;*

*SCS = FSC + WSC if FSC != NULL AND WSC != NULL;*

In the Framingham Offspring Cohort, we identified total family income for the indicator of the financial strain component (FSC); job insecurity and physiological job demand scale for the indicators of work related difficulties component (WSC); marital disagreement for the indicator of relationship or marital problems (MSC); and spouse’s heart attack, stroke and heart disease-related death for the indicators of serious health problems of spouse (HOSC) [6, 7]. The pseudocode for Framingham is below:

*WSC = 1 if JOB_INSEC==HIGH AND JOB_DEMAND == HIGH;*

*WSC = 0 if JOB_INSEC==LOW OR JOB_DEMAND == LOW;*

*MSC = 1 if MARITAL_DISAGR == HIGH;*

*MSC = 0 if MARITAL_DISAGR == LOW;*

*FSC = 1 if FAM_INCOME == LOW;*

*FSC = 0 if FAM_INCOME == HIGH;*

*HOSC = 1 if spouse had heart attack, stroke, and heart disease-related death;*

*HOSC = 0 if spouse did not have one of the above problem;*

*SCS = MSC+FSC+WSC+HOSC if no more than one component missing*

**SNP Quality Control:**

These QCs included minimum minor allele frequency (MAF) of 0.01 (1%), maximum missing genotypes of 0.05 (5%) per individual, maximum missing individuals per SNP of 0.05 (5%), and significant departure from Hardy-Weinberg equilibrium (HWE) (*p*<0.00001). We also checked for misclassification for sex, cryptic relatedness (as implemented in PLINK) or the same family ID suggesting a potential for non-independent observations. In such cases, we included only one individual from a family in the phenotype files for analysis.

**Coding for Studies from Multiple Sources:**

Meta-analysis requires that the study source be taken into account when generating a summary estimate across those studies. This allows for the assessment of between-study heterogeneity, that is, how stable the final summary estimate is across studies. This accounting is usually achieved by estimating a model with either random effects or fixed effects. Random effects, in this case random intercepts, specify study source in a model as a “clustering” variable, allowing the intercepts in the model to be different for each study. In contrast, in the fixed effects model study source is accounted for by including dummy variables that represent the study source. There is some debate over which approach to use under a given set of circumstance. Typically, when there are not many datasets (clusters), the dummy variable approach is generally deemed acceptable. Dummy variable coding is a standard statistical practice when needing to incorporate a categorical variable with two or more categories in the model. On the other hand, especially when there is considerable between-study variability in the association under study, the random effects model may be preferable. In the present case, there were relatively few studies, but there was considerable between-study variability. In the main article, we elected to report the summary estimate of correlation derived from the fixed effects model (Table 1) because it was smaller in magnitude (0.27) and thus more conservative than the summary estimate derived from the random effects model (0.32). The coding for the 11 dummy variables for the 12 studies is given below, where the last study “ARIC” is the reference:

x1 x2 x3 x4 x5 x6 x7 x8 x9 x10 x11

CARDIA 1 0 0 0 0 0 0 0 0 0 0

FRAM 0 1 0 0 0 0 0 0 0 0 0

JHS 0 0 1 0 0 0 0 0 0 0 0

MESA 0 0 0 1 0 0 0 0 0 0 0

WHI 0 0 0 0 1 0 0 0 0 0 0

CARE 0 0 0 0 0 1 0 0 0 0 0

CHASE 0 0 0 0 0 0 1 0 0 0 0

DFHS 0 0 0 0 0 0 0 1 0 0 0

STR1 0 0 0 0 0 0 0 0 1 0 0

STR2 0 0 0 0 0 0 0 0 0 1 0

STRP 0 0 0 0 0 0 0 0 0 0 1

ARIC 0 0 0 0 0 0 0 0 0 0 0

**Outlier Detection and Removal:**

Although there are a variety of approaches to managing outliers [17], such as identifying values that are 3 standard deviations from the mean or below the 1st percentile and above the 99th percentile, and either treating that value as missing, or recoding it to a less extreme value. These typically involve making decisions about values that are biologically plausible but more extreme than the majority of data points. In our case, the nature of the data made the process relatively simple, as there were clear cases where values were quite far out in the tails of the distributions, away from virtually all other cases. These cases were simply excluded in our analysis. For example, in Table S3 the maximum value of insulin 6672.75 and minimum value for LDL 0 in the following table clearly appear to be outliers. Also, one potential challenge in this regard was that it is not always possible to determine whether outlying values are data errors or real data.

**Additional Discussion:**

***Need for Data Harmonization:***

The influence of psychosocial stress on human health conditions, particularly CVD, has been studied extensively [18, 19] and many approaches have been developed to assess psychosocial stress [8, 10]; however, there are a large number of studies that did not have a self-rated formal stress measure. The construction of a synthetic chronic psychosocial stress measure in such datasets, a key innovation in our work, will enable investigators to evaluate the role of psychosocial stress on human health in a large pool of datasets. Although efforts like the PhenX Project [20] represent significant progress toward standardizing study data measures for various domains of epidemiological and genomic research for new studies, the need for harmonization of extant datasets remains. The ideal case would be for the biomedical research community to adopt uniform standards in defining, measuring and quantifying human health conditions and risk factors.

***Harmonization Steps and Alternative Approaches:***

The increased interest in harmonizing individual participant level data is relatively recent. Consequently, the area is still evolving and there are a number of open questions regarding the best approaches. Some decisions arise out of necessity—for example, the selection of datasets depends on whether the dataset is available at all for sharing, and if so, how well the available variables are suited to the research question. From there, it is a matter of evaluating how best to code or transform variables in order to make them consistent across the datasets. This process ranges from simple standardization of coding (e.g., male=”M”;female=”F”) or converting to a common metric, for example, ensuring that all values of total serum cholesterol are either in mg/dL or International Units.

Identification and removal of inconsistencies of codings and units can be a cumbersome process but it is relatively less complex compared to making up for the missing variables, which can be done using imputation or by deriving values from proxy variables. In our project, the variable that required the most work along these lines was the synthetic stress measure. Imputation has more often been used in making-up missing values of a variable than making up whole variable. A combination of both approaches can also be used to maximize the information, if there are missing values in a derived variable. In order to construct the missing variable of chronic psychosocial stress, we have used our algorithm [6] to derive the variable using proxy indicators of stress and, as described in the Limitation Section, we have so far chosen not to impute for missing variables and indicators in our approach. This choice was supported by our prior work [6] showing that the stress scores which include less than the full set of indicators still behave similarly in terms of associations with other phenotypes, such as depressive symptoms. In this systematic analysis, we observed polychoric correlations between the self‐rated score and synthetically derived scores using worst case two, three, and all four available components and respective Rho were 0.15, 0.19, and 0.23, which were close and suggested that the synthetic score developed using incomplete set of inferred indicators could still be useful. Although our approach offers a utilitarian solution in this case, there are more sophisticated approaches to managing missing data, such as integrative data analysis [21] or multiple imputation methods [22]. In the end, the selection of one of these approaches is both an empirical and philosophical one. Philosophically, the simplest sufficient technique is always preferred. The empirical results determine whether that simplest choice is sufficient. In the present case, the results described above convinced us that our approach was sufficient. Another example of making choice from alternate options was choosing a method to calculate the summary estimate of correlation in Table 1 using fixed effects model vs. random effects model. We used the fixed effect model as it provided smaller estimate in magnitude (0.27) and thus more conservative than the summary estimate derived from the random effects model (0.32).

***Insights from Large Sample Resulting from Combining the Datasets:***

A perennial challenge in conducting medical or behavioral research is that studies of the same phenomenon can differ greatly in their conclusions. Many of these differences can be attributed to large or even subtle differences in study design and the context and composition of the samples. Moreover, it has long been recognized that when effect sizes are relatively small, such as single variant genetic studies, many studies are not of sufficient sample size to conduct an adequate null hypothesis test or to generate an estimate that is close to the true population value (i.e., small sample sizes render estimates with large standard errors). Meta-analysis in general was developed to address precisely this problem. Summarizing results over multiple studies, either through conventional meta-analysis, or as in our case using a dataset that has the individual participant data from each study to conduct a mega-analysis—is thought to produce a more robust estimate of the association under study, and potentially more generalizable insights [23].

Table S1: Unit conversion formulas and factors

| Measurement | SI Unit | Other Unit(s) | Conversion |
| --- | --- | --- | --- |
| Insulin | pmol/L | uU/mL | pmol/L = 6.945* uIU/ml |
|  |  | uIU/ml | pmol/L= 6.945 * mU/L |
|  |  | mIU/L | mIU/L = uIU/ml = uU/ML |
| Glucose | mmol/L | mg/d | mmol/L = 0.05551 * mg/dL |
|  |  | mg/100 ML | MG/100 ML = mg/dL |
| Total Cholesterol | mmol/L | mg/dL | mmol/L = 0.02586 * mg/dL |
| LDL Cholesterol | mmol/L | mg/dL | mmol/L = 0.02586 * mg/dL |
| HDL Cholesterol | mmol/L | mg/dL | mmol/L = 0.02586 * mg/dL |
| Triglycerides | mmol/L | mg/dL | mmol/L = 0.01129 * mg/dL |
| Stress |  |  | Ordinal score to z-score |
| Depressive symptoms |  |  | Ordinal score to z-score |

Table S2 (Panel A): Data variable harmonization chart for dbGaP public-access datasets. The empty cells represent missing data. The unit codes as shown in upper or lower case for each study as they were recorded in the dataset.

| Phenotypes | Harmonized _UNIT | MESA_CODE/UNIT | FRAM_CODE/UNIT | ARIC_CODE/UNIT | CARDIA_CODE/UNIT | WHI_CODE/UNIT | JHS_CODE/UNIT |
| --- | --- | --- | --- | --- | --- | --- | --- |
| Sex | 0=Female, 1=Male | 1:Male, 2:Female | 1=Male, 2=Female | 1=Male, 2=Female | 1:Male, 2:  Female | 2:Female | 1:Male, 2:FEMALE |
| Race | W=White, B=Black, H=Hispanic, A=Asian, N=Native American | 1:White, 2:Chinese-American, 3:Blacks, 4:Hispanic | 1=White, 2=Black, 3=Native Amer, 4=Asian, 5=Hispanic | B=Black, W=White | 3:Hispanic, 4:Black,  5:White | 1:American Indian, 2:Asian, 3:Black, 4:Hispanic, 5:White, 8:Other | African American |
| Age | Years | Years | Years | Years | Years | Years | Years |
| Systolic BP | mm/Hg | mm Hg | MM HG | mm Hg | mm Hg | mm Hg | mm Hg |
| Diastolic BP | mm/Hg | mmHg | MM HG | mm Hg | mmHg | mmHg | mm Hg |
| Body Mass Index | kg/m2 | kg/m2 | kg/m2 | Kg/(m2) | kg/m2 | kg/m2 | kg/m2 |
| Waist Circumference | cm | cm | IN | cm | cm | cm | cm |
| Hip Circumference | cm | cm | IN | cm | cm | cm |  |
| Fasting Glucose | mmol/L | mg/dL | mg/Dl | mg/dL | MG/100 ML | Mg/dl | mg/dl |
| HBA1c | % | % | % |  |  |  |  |
| Insulin | pmol/L | umol/L, mU/L | PMOL/L | pmol/L | uU/ML | uIU/ml | uU/mL |
| Type II Diabetes | 0=no;1=yes | 0-Normal, 1-Untreated+Treated | 0=No, 1=Yes | 0:No, 1:Yes | 1:NO, 2:YES, 8:Not sure | 0=No, 1=Yes | 0:No, 1: Yes, X: Don’t Know |
| Total Cholesterol | mmol/L | mg/dl | Mg/100 ml | mg/dL | MG/DL | Mg/dl | mg/dl |
| Low Density Lipoprotein | mmol/L | mg/dl | mg/dl | mg/dL | MG/DL | Mg/dl | mg/dl |
| High Density Lipoprotein | mmol/L | mg/dl | mg/dl | mg/dL | MG/DL | Mg/dl | mg/dl |
| Triglycerides | mmol/L | mg/dl | mg/Dl | mg/dl | MG/DL | Mg/dl | mg/dl |
| Depressive Symptoms Instrument | Z-Score | CES-D, score 0-53 | CES-D, score 0 -0.8505 | Maastricht Vital Exhaustion, score 0-42 | CES-D, score 0-52 | Shortened CES-D, score 0.00028-0.95938 | CES-D, score 0-47 |
| Psychosocial Stress | Z-Score | Self-rated, score 0 - 5 | Synthetic, score 0-4 | Synthetic, score 0-3 | Synthetic, score 0-3 | Synthetic 0-5 | Self-rated, score 0-23 |

Table S2 (Panel B): Data variable harmonization chart for Duke datasets. The empty cells represent missing data. The unit codes as shown in upper or lower cases for each study as they were recorded in the dataset.

| Phenotypes | Harmonized CODE/UNIT | CHASE CODE/UNIT | DFHS CODE/UNIT | CAREGIVER CODE/UNIT | STRRIDE-1 CODE/UNIT | STRRIDE-AT/RT CODE/UNIT | STRRIDE-PD CODE/UNIT |
| --- | --- | --- | --- | --- | --- | --- | --- |
| Sex | 0=Female, 1=Male | M:Male, F:Female | M:Male, F:Female | M:Male, F:Female | M:Male, F:Female | M:Male,  F:Female | M:Male, F:Female |
| Race | W=White, B=Black, H=Hispanic, A=Asian, N=Native American" | W=White, B=Black | 1=White, 2=Black | W=White, B=Black | A=Asian; B=Black; H=Hispanic; I=Indian; W=Whites | A=Asian; AA=African American; H=Hispanic; I=Indian; C=Caucasian | A=Asian; AA=African American; H=Hispanic; I=Indian; C=Caucasian |
| Age | Years | Years | Years | Years | Years | Years | Years |
| Systolic BP | mm/Hg | mm/Hg | mm/Hg | mm/Hg | mm/Hg | mm/Hg | mm/Hg |
| Diastolic BP | mm/Hg | mm/Hg | mm/Hg | mm/Hg | mm/Hg | mm/Hg | mm/Hg |
| Body Mass Index | kg/m2 | kg/m2 | Kg/m2 | kg/m2 | Kg/(m2) | kg/m2 | kg/m2 |
| Waist Circumference | cm |  | cm | cm | cm | cm | cm |
| Hip Circumference | cm |  | cm | cm | cm | cm | cm |
| Fasting Glucose | mmol/L | mmol/L | mmol/L | mmol/L | mmol/L | mmol/L | mmol/L |
| HBA1c | % |  | mmol/L | mmol/L |  |  | mmol/L |
| Insulin | pmol/L | pmol/L | pmol/L | pmol/L | pmol/L | pmol/L | pmol/L |
| Type II Diabetes | 0=No, 1=Yes |  |  | 0=No, 1=Yes |  |  |  |
| Total Cholesterol | mmol/L | mmol/L | mmol/L | mmol/L | mmol/L | mmol/L | mmol/L |
| Low Density Lipoprotein | mmol/L | mmol/L | mmol/L | mmol/L | mmol/L | mmol/L | mmol/L |
| High Density Lipoprotein | mmol/L | mmol/L | mmol/L | mmol/L | mmol/L | mmol/L | mmol/L |
| Triglycerides | mmol/L | mmol/L | mmol/L | mmol/L | mmol/L | mmol/L | mmol/L |
| Depressive Symptom Instrument | Z-Score | BDI | CES-D | CES-D |  | Self-rated | Self-rated |
| Psychosocial Stress | Z-Score | Synthetic, score 0-3 | Synthetic, score 0-2 | Synthetic, score 0-5 |  | Synthetic, score 0-2 | Synthetic, score 0-2 |

Table S3: Summary statistics of harmonized CVD-risk variables.

| Variable | N | Mean | SD | Minimum | Maximum |
| --- | --- | --- | --- | --- | --- |
| Age, years | 36,181 | 54.0 | 12.4 | 17 | 89 |
| Systolic Blood Pressure, mmHg | 35,753 | 123.7 | 18.9 | 61 | 242 |
| Diastolic Blood Pressure, mmHg | 35,744 | 74.5 | 10.8 | 12 | 144 |
| Body Mass Index, Kg/M^2^ | 35,944 | 28.3 | 5.8 | 10.8 | 75.0 |
| Waist Circumference, cm | 35,536 | 93.4 | 15.0 | 30.0 | 244.0 |
| Hip Circumference, cm | 34,412 | 105.6 | 11.5 | 38.0 | 192.0 |
| Fasting Glucose, mmol/L | 26,534 | 5.6 | 1.9 | 1.9 | 34.9 |
| HbA1c, % | 9,251 | 5.6 | 1.0 | 3.0 | 15.9 |
| Insulin, pmol/L | 27,074 | 90.7 | 142.5 | 6.9 | 6672.7 |
| Total Cholesterol, mmol/L | 27,619 | 5.3 | 1.07 | 1.2 | 15.4 |
| Low Density Lipoprotein, mmol/L | 26,976 | 3.3 | 1.0 | 0 | 13.0 |
| High Density Lipoprotein, mmol/L | 27,603 | 1.3 | 0.4 | 0.2 | 4.2 |
| Triglycerides, mmol/L | 27,620 | 1.4 | 1.0 | 0.1 | 44.2 |
| Depressive symptoms, z-scores | 33,749 | 0 | 1 | -1.4 | 6.1 |
| Synthetic Stress Scale, raw scores | 35,310 | 1.2 | 1.5 | 0 | 21 |
| Synthetic Stress Scale, z-scores | 35,310 | 0 | 1 | -1.4 | 4.3 |

Table S4: Harmonization of candidate SNPs that were found to moderate association of stress with endophenotypes in pathways to CVD across the multiple studies to be included in a mega-analysis.

| Candidate SNP | Present in all studies? | LD SNP | $R^{2}$ | Minor Allele | Overall MAF | Whites MAF | Blacks MAF | HWE P-value  across all datasets |
| --- | --- | --- | --- | --- | --- | --- | --- | --- |
| *EBF1* rs4704963 | NO | rs17056278 | 1.0 | C | 0.05 | 0.07 | 0.02 | 0.04 - 1.0 |
| *5HTR2C* rs6318 | NO | rs2428722, rs2428730 | 1.0 | C | 0.24 | 0.17 | 0.36 | 0.10 - 0.83 |
| *BDNF* rs6265 | YES | - | - | T | 0.18 | 0.19 | 0.04 | 0.0009 - 0.81 |

**References:**

1. The WHI Study Group: Design of the Women’s Health Initiative Clinical Trial and Observational Study. *Controlled Clinical Trials* 1998, 19(1):61-109.

2. Friedman GD, Cutter GR, Donahue RP, Hughes GH, Hulley SB, Jacobs DR *et al*: Cardia: study design, recruitment, and some characteristics of the examined subjects. *Journal of Clinical Epidemiology* 1988, 41(11):1105-1116.

3. The ARIC Investigators: THE ATHEROSCLEROSIS RISK IN COMMUNIT (ARIC) STUI)Y: DESIGN AND OBJECTWES. *American Journal of Epidemiology* 1989, 129(4):687-702.

4. Feinleib M, Kannel WB, Garrison RJ, McNamara PM, Castelli WP: The framingham offspring study. Design and preliminary data. *Preventive Medicine* 1975, 4(4):518-525.

5. Bild DE, Bluemke DA, Burke GL, Detrano R, Diez Roux AV, Folsom AR *et al*: Multi-Ethnic Study of Atherosclerosis: Objectives and Design. *American Journal of Epidemiology* 2002, 156(9):871-881.

6. Singh A, Babyak MA, Brummett BH, Jiang R, Watkins LL, Barefoot JC *et al*: Computing a Synthetic Chronic Psychosocial Stress Measurement in Multiple Datasets and its Application in the Replication of G × E Interactions of the EBF1 Gene. *Genetic Epidemiology* 2015, 39(6):489-497.

7. Singh A, Babyak MA, Nolan DK, Brummett BH, Jiang R, Siegler IC *et al*: Gene by stress genome-wide interaction analysis and path analysis identify EBF1 as a cardiovascular and metabolic risk gene. *Eur J Hum Genet* 2015, 23(6):854-862.

8. Shivpuri S, Gallo LC, Crouse JR, Allison MA: The Association Between Chronic Stress Type and C-Reactive Protein in the Multi-Ethnic Study of Atherosclerosis (MESA): Does Gender Make a Difference? *Journal of Behavioral Medicine* 2012, 35(1):74-85.

9. Sempos CT, Bild DE, Manolio TA: Overview of the Jackson Heart Study: A Study of Cardiovascular Diseases in African American Men and Women. *The American Journal of the Medical Sciences* 1999, 317(3):142-146.

10. Johnson DA, Lisabeth L, Lewis TT, Sims M, Hickson DA, Samdarshi T *et al*: The Contribution of Psychosocial Stressors to Sleep among African Americans in the Jackson Heart Study. *Sleep* 2016, 39(7):1411-1419.

11. Burroughs AR, Visscher WA, Haney TL, Efland JR, Barefoot JC, Williams RB *et al*: Community Recruitment Process by Race, Gender, and SES Gradient: Lessons Learned from the Community Health and Stress Evaluation (CHASE) Study Experience. *Journal of Community Health* 2003, 28(6):421-437.

12. Slentz CA, Aiken LB, Houmard JA, Bales CW, Johnson JL, Tanner CJ *et al*: Inactivity, exercise, and visceral fat. STRRIDE: a randomized, controlled study of exercise intensity and amount. *Journal of Applied Physiology* 2005, 99(4):1613-1618.

13. Slentz CA, Bateman LA, Willis LH, Shields AT, Tanner CJ, Piner LW *et al*: Effects of aerobic vs. resistance training on visceral and liver fat stores, liver enzymes, and insulin resistance by HOMA in overweight adults from STRRIDE AT/RT. *American Journal of Physiology - Endocrinology And Metabolism* 2011, 301(5):E1033.

14. Slentz CA, Bateman LA, Willis LH, Granville EO, Piner LW, Samsa GP *et al*: Effects of exercise training alone vs a combined exercise and nutritional lifestyle intervention on glucose homeostasis in prediabetic individuals: a randomised controlled trial. *Diabetologia* 2016, 59(10):2088-2098.

15. Siegler IC, Brummett BH, Williams RB, Haney TL, Dilworth-Anderson P: Caregiving, residence, race, and depressive symptoms. *Aging & mental health* 2010, 14(7):771-778.

16. Brummett BH, Boyle SH, Ortel TL, Becker RC, Siegler IC, Williams RB: Associations of depressive symptoms, trait hostility, and gender with C-reactive protein and interleukin-6 response following emotion recall. *Psychosomatic medicine* 2010, 72(4):333-339.

17. Ord K: Outliers in statistical data : V. Barnett and T. Lewis, 1994, 3rd edition, (John Wiley & Sons, Chichester), 584 pp., [UK pound]55.00, ISBN 0-471-93094-6. *International Journal of Forecasting* 1996, 12(1):175-176.

18. Kamarck T: Psychosocial stress and cardiovascular disease: An exposure science perspective. *Psychological Science Agenda* 2012, 26.

19. Lazarus RS: Psychological stress and the coping process. 1966.

20. Hamilton CM, Strader LC, Pratt JG, Maiese D, Hendershot T, Kwok RK *et al*: The PhenX Toolkit: Get the Most From Your Measures. *American Journal of Epidemiology* 2011, 174(3):253-260.

21. Curran PJ, Hussong AM: Integrative data analysis: The simultaneous analysis of multiple data sets. *Psychological Methods* 2009, 14(2):81-100.

22. Sterne JAC, White IR, Carlin JB, Spratt M, Royston P, Kenward MG *et al*: Multiple imputation for missing data in epidemiological and clinical research: potential and pitfalls. *BMJ* 2009, 338.

23. Ioannidis JPA, Rosenberg PS, Goedert JJ, O’Brien TR: Commentary: Meta-analysis of Individual Participants’ Data in Genetic Epidemiology. *American Journal of Epidemiology* 2002, 156(3):204-210.
